# Microbial Detoxification of Bifenthrin by a Novel Yeast and Its Potential for Contaminated Soils Treatment

**DOI:** 10.1371/journal.pone.0030862

**Published:** 2012-02-13

**Authors:** Shaohua Chen, Jianjun Luo, Meiying Hu, Peng Geng, Yanbo Zhang

**Affiliations:** Laboratory of Insect Toxicology, Key Laboratory of Pesticide and Chemical Biology, Ministry of Education, South China Agricultural University, Guangzhou, People's Republic of China; University of Groningen, The Netherlands

## Abstract

Bifenthrin is one the most widespread pollutants and has caused potential effect on aquatic life and human health, yet little is known about microbial degradation in contaminated regions. A novel yeast strain ZS-02, isolated from activated sludge and identified as *Candida pelliculosa* based on morphology, API test and 18S rDNA gene analysis, was found highly effective in degrading bifenthrin over a wide range of temperatures (20–40°C) and pH (5–9). On the basis of response surface methodology (RSM), the optimal degradation conditions were determined to be 32.3°C and pH 7.2. Under these conditions, the yeast completely metabolized bifenthrin (50 mg·L^−1^) within 8 days. This strain utilized bifenthrin as the sole carbon source for growth as well as co-metabolized it in the presence of glucose, and tolerated concentrations as high as 600 mg·L^−1^ with a *q*_max_, *K*_s_ and *K*_i_ of 1.7015 day^−1^, 86.2259 mg·L^−1^ and 187.2340 mg·L^−1^, respectively. The yeast first degraded bifenthrin by hydrolysis of the carboxylester linkage to produce cyclopropanecarboxylic acid and 2-methyl-3-biphenylyl methanol. Subsequently, 2-methyl-3-biphenylyl methanol was further transformed by biphenyl cleavage to form 4-trifluoromethoxy phenol, 2-chloro-6-fluoro benzylalcohol, and 3,5-dimethoxy phenol, resulting in its detoxification. Eventually, no persistent accumulative product was detected by gas chromatopraphy-mass spectrometry (GC-MS) analysis. This is the first report of a novel pathway of degradation of bifenthrin by hydrolysis of ester linkage and cleavage of biphenyl in a microorganism. Furthermore, strain ZS-02 degraded a variety of pyrethroids including bifenthrin, cyfluthrin, deltamethrin, fenvalerate, cypermethrin, and fenpropathrin. In different contaminated soils introduced with strain ZS-02, 65–75% of the 50 mg·kg^−1^ bifenthrin was eliminated within 10 days, suggesting the yeast could be a promising candidate for remediation of environments affected by bifenthrin. Finally, this is the first described yeast capable of degrading bifenthrin.

## Introduction

Synthetic pyrethroid insecticides are frequently and widely used worldwide. These kinds of pesticides are synthetic analogs of the naturally occurring toxin, derived from the flowers of Chrysanthemum cinerariaefolium [1]. They have been classified as type I or type II based on the toxic symptoms and the absence or presence of a cyano group at the carboxyl alpha position. Recently, with the restriction on the use of organophosphates and carbamates, pyrethroids have generally been regarded as the replacements [2]. At present, they are the dominant insecticides among retail sales to consumers [3], and their usage is expected to further increase [4], [5]. Due to these uses, pyrethroids have been reported in nearly all sediment samples tested from urban creeks [2], [3], [6], [7], [8].

The accumulation and widespread use of pyrethroids in agriculture have increased the public concern on potential human health risks that may result from chronic dietary exposure to pyrethroid residues on food [9], [10]. For instance, numerous studies have shown that they may have neurotoxicity [11], [12], immunotoxicity [13], [14], reproductive toxicity [5], [15], and genotoxicity [16], [17] on non-target organisms. In addition, some current-use pyrethroids have recently been listed as potential endocrine disruptors by the Environmental Protection Agency (EPA) of the USA [18]. All these findings indicated that pyrethroids might be potentially harmful to human health. It is thus critically essential to develop an efficient and cost-effective remediation strategy to eliminate pyrethroids from the environments and agricultural products.

Bifenthrin[2-methylbiphenyl-3-ylmethyl-(Z)-(1RS)-3-(2-chloro-3,3,3-trifluoroprop-1-enyl)-2,2-dimethylcyclopropane carboxylate] is one of the most popular pyrethroids effective against a broad spectrum of insect pests of economically important crops [5], [16]. It is also extensively used for the control of residential pests such as termites in urban areas [19]. Its half-life in soil is usually between 65 and 125 days, but can range from 2 weeks to over 1 year, depending on the soil type, moisture, pH, temperature, and other conditions [20]–[22]. The World Health Organization (WHO) has classified bifenthrin as Toxicity Class II moderately hazardous [23]. In urban sediments, bifenthrin contributed the most to the observed toxicity among pyrethroids [2], [6], [8]. Therefore, bifenthrin was the pyrethroid of greatest concern with regards to aquatic life toxicity and widespread occurrence in water and soil [2], [8].

Bifenthrin released to surface waters or sediments is subjected to hydrolysis, photodecomposition, volatilization, and aerobic degradation by microorganisms [20]. Microbial degradation is considered to be the most significant process determining the fate of bifenthrin and other pyrethroids in nature [24], and has received increasing attention as an effective, cheap, and safe approach to clean up contaminated environments [25]. To date, some pyrethroid-degrading microorganisms such as *Bacillus cereus* [26], *Pseudomonas fluorescens* [27], *Micrococcus* sp. CPN 1 [28], *Sphingobium* sp. JZ-2 [29], *Serratia* sp. JCN13 [30], *Ochrobactrum tritici* pyd-1 [31], and *Cladosporium* sp. HU [32] have been isolated. However, there was little information available on bifenthrin-degrading microorganism.

In the present study, a novel yeast *Candida pelliculosa* ZS-02 capable of degrading bifenthrin was isolated and characterized. The objective of this study was to optimize its degradation conditions, investigate its degradation pathway and evaluate its potential in bioremediation of bifenthrin-contaminated soils. Finally, obtained information illustrated that the isolated strain might have potential for use in bioremediation of bifenthrin-contaminated environments.

## Materials and Methods

### Chemicals and media

Technical grade bifenthrin (98% purity), cyfluthrin (95% purity), deltamethrin (98% purity), fenvalerate (91.2% purity), cypermethrin (92.9% purity), and fenpropathrin (93% purity) used in this study were obtained from Zhongshan Aestar Fine Chemical Inc., Ltd, China. Chromatographic grade acetonitrile were purchased from Sigma-Aldrich, USA. All other chemicals and solvents were purchased from Merck, Germany.

The mineral salt medium (MSM) containing (grams per liter) (NH_4_)_2_SO_4_, 2; MgSO_4_·7H_2_O, 0.2; CaCl_2_·2H_2_O, 0.01; FeSO_4_·7H_2_O, 0.001, Na_2_HPO_4_·12H_2_O, 1.5; and KH_2_PO_4_, 1.5; and yeast peptone dextrose (YPD) medium containing (grams per liter) yeast extract, 10; peptone, 20; and dextrose (or glucose), 20 were used for the isolation and cultivation of bifenthrin-degrading yeast, respectively. The final pH was adjusted to 7.2. Both media were autoclaved for 20 min at 121°C separately.

### Isolation and screening of bifenthrin-degrading microorganisms

Activated sludge samples were collected as the inoculum from an aerobic pyrethroid-manufacturing wastewater treatment system located in Zhongshan (Guangdong, China), which had produced pyrethroids for many years. 30-mL of activated sludge was transferred into 250-mL Erlenmeyer flasks containing 50 mL sterilized MSM enrichment medium. Bifenthrin dissolved in acetone solution was added to a final concentration of 50 mg·L^−1^ as the sole carbon source. The enrichment culture was incubated for 7 days at 30±1°C with shaking at 150 rpm. A 5-mL from each enrichment culture was transferred into 50 mL of fresh enrichment medium containing 100 mg·L^−1^ of bifenthrin and incubated for another 7 days. Three additional successive transfers were made into media containing 200, 400, and 600 mg·L^−1^ of bifenthrin. The final cultures were serially diluted and spread on YPD agar plates. The plates were incubated for 5 days at 30°C, and colonies were picked and purified by re-streaking 3 times as described by Chen et al. [32], [33]. The abilities of isolates to degrade bifenthrin were determined by high performance liquid chromatography (HPLC) (Agilent, USA) according to Chen et al. [34].

### Characterization and identification of the bifenthrin-degrading isolates

One bifenthrin-degrading isolate that showed highest degradation efficiency was selected for further study. The isolate was grown on YPD agar plates at 30°C for 5 days and its morphology was investigated with a light microscope (Olympus, Japan) and scanning electron microscope (XL-30 ESEM, Philips Optoelectronics Co., Ltd, Holland). Colony morphology was observed on YPD agar plates incubated at 30°C at 1, 3, 5, and 7 days according to Barnett et al. [35]. The isolate was also subjected to sugar fermentation pattern analysis using API 20C AUX system (bioMérieux, France) according to the instructions of the manufacturer.

The isolate was confirmed by 18S rDNA sequence analysis. Total genomic DNA was prepared according to standard methods [36]. The 18S rDNA gene was amplified with the yeast universal primers EF4 (5′–GGAAGGGRTGTATTTATTAG–3′) and EF3 (5′–TCCTAAATGACCAAGTTTG–3′) [35]. Amplification was carried out in 50 µL reaction mixture containing 5 µL of 10×Ex *Tap* reaction buffer, 1 µL of 2.5 mmol·L^−1^ dNTP, 1 µL of 10 µmol·L^−1^ each primer, 1 µL of genomic DNA, 0.5 µL of 5 U·µL^−1^ Ex *Tap* DNA polymerase and 40.5 µL of ultrapure water. Reaction conditions consisted of initial denaturation at 94°C for 5 min, followed by 35 cycles of denaturation at 94°C for 1 min, annealing at 48°C for 1 min, and extension at 72°C for 2 min, with the last cycle followed by a ten-minute extension at 72°C. Polymerase chain reaction (PCR) product containing the amplified 18S rDNA gene fragment was purified with QIAquick Gel Extraction Kit spin column (Guangzhou Heda Technology Co. Ltd., China), ligated to the linear vector pMD20-T (TaKaRa Biotechnology Co. Ltd., China), and transformed into competent *Escherichia coli* DH5α cells. Positive clones were screened and sent to Shanghai Invitrogen Technology Co. Ltd., China, for sequencing. The 18S rDNA gene sequences with 1,452 bp were deposited at the GenBank under the accession number JN700989. The resulting sequence was compared with the genes available in the GenBank nucleotide library by a BLAST search through the National Center for Biotechnology Information (NCBI) internet site. Multiple alignments of 18S rDNA were carried out using CLUSTALX 1.8.1 software, and phylogenesis was analyzed using MEGA 4.0 software. An unrooted tree was built using the neighbor-joining method [37].

### Inoculum preparation

To prepare the inoculum, the pure culture, obtained from individual colonies, was grown in YPD medium for 5 days, harvested by centrifugation at 4000×*g* for 5 min, washed twice with 0.9% sterile normal saline and re-suspended in MSM to set an OD_600_ of 0.3 by a spectrophotometer (Shimadzu, Japan). One percent of this suspension (1.0×10^7^ CFU·mL^−1^) was used as the inoculum for studies.

### Optimization of the bifenthrin-degrading conditions

The preliminary study indicated that temperature, pH and inoculum were significant variables for the degradation of bifenthrin by the yeast. In single-factor experiments, we determined the optimal ranges of the three factors, temperature: 20–40°C, pH: 5–9, and inoculum: OD_600_ = 0.1–0.5. These specified ranges were used as the reference levels. Furthermore, response surface methodology (RSM) based on the Box-Behnken design was used to optimize the main and interactive effects of the important parameters which significantly influenced the bifenthrin degradation by the yeast [38]. A three-variable Box-Behnken design consisting of 15 experimental runs with three replicates at the center point was generated by statistic analysis system (SAS) software (Version 9.0). The symbols and levels of three independent variables are shown in Table 1. The dependent variable was the degradation of 50 mg·L^−1^ bifenthrin in MSM by the yeast after 5 days of culture. Experiment was conducted according to Randomized block design and data were analyzed by response surface regression procedure of the SAS software to fit the following quadratic polynomial equation (Eq.(1)).
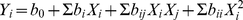
(1)where *Y*_i_ is the predicted response, *X*_i_ and *X*_j_ are variables, *b*_0_ is the constant, *b*_i_ is the linear coefficient, *b*_ij_ is the interaction coefficient, and *b*_ii_ is the quadratic coefficient.

**Table 1:** Box-Behnken experimental design and the response of dependent variable for bifenthrin degradation.

				Response
Run	*X*_1_	*X*_2_	*X*_3_	Bifenthrin degradation (%)*Y*_ZS-02_
1	−1	−1	0	55.5±1.5o
2	−1	1	0	65.2±0.6n
3	1	−1	0	67.6±0.3m
4	1	1	0	74.1±0.8i
5	0	−1	−1	72.3±0.5j
6	0	−1	1	75.5±0.4g
7	0	1	−1	75.2±0.4h
8	0	1	1	77.8±0.2f
9	−1	0	−1	68.4±1.2l
10	1	0	−1	79.5±0.4e
11	−1	0	1	70.6±0.5k
12	1	0	1	80.3±0.5d
13	0	0	0	86.5±0.4a
14	0	0	0	85.3±0.6b
15	0	0	0	83.1±1.0c

*X*_1_: temperature, −1 (20°C), 0 (30°C), +1 (40°C); *X*_2_: pH, −1 (5), 0 (7), +1 (9); *X*_3_: inoculum, −1 (OD_600_ = 0.1), 0 (OD_600_ = 0.3), +1 (OD_600_ = 0.5). The data presented are means of three replicates with standard deviation, which is within 5% of the mean. Different letters indicate significant differences (*p*<0.05, LSD test).

### Biodegradation of bifenthrin in liquid medium

Growth experiments with bifenthrin as the sole carbon source were performed in 250-mL Erlenmeyer flasks containing 50 mL sterile MSM with 50 mg·L^−1^ bifenthrin. Co-metabolism experiments were carried out in the same medium supplemented with 1% (*w*/*v*) glucose [39]. The seed suspension was aseptically inoculated into the MSM and incubated for 7 days at pH 7.2 at 32°C with shaking at 150 rpm. Each treatment was set in triplicate with non-inoculated samples as control. Samples (30-mL) were withdrawn periodically from the cultures to examine growth by recording the optical density (OD) value at 600 nm using spectrophotometer and to measure the bifenthrin concentration by HPLC as described previously [34], [40].

Biodegradation experiments by the yeast with different concentrations of bifenthrin (100–600 mg·L^−1^) and with various pyrethroids including bifenthrin, cyfluthrin, deltamethrin, fenvalerate, cypermethrin, and fenpropathrin (50 mg·L^−1^) were carried out in MSM supplemented with 1% glucose for 5 days at pH 7.2 at 32°C.

### Identification of bifenthrin metabolites

The metabolites of bifenthrin in cell-free filtrates of the yeast cultures grown in MSM containing 50 mg·L^−1^ of bifenthrin were detected by gas chromatopraphy-mass spectrometry (GC-MS) (Agilent, USA). The cell-free filtrates were collected at 2, 4, 6, and 8 days, respectively. After acidification to pH 2 with 2 M HCl, the cultures were extracted with ethyl acetate and supernatant was dehydrated, dried and re-dissolved in methanol [28]. After filtration with 0.45 µm membrance (Millipore, USA), the samples were analyzed by GC-MS according to Zhang et al. [40].

### Biodegradation of bifenthrin in soil

Soil samples were taken from a depth of 5–20 cm over a 25 m^2^ area at grass-covered field, sieved to 2 mm and stored at 4°C in the dark for 14 days, before use [24]. The physicochemical properties of the soil were (grams per kilogram of dry weight): organic matter, 10.5; total N, 0.5; total P, 0.4; total K, 18.2; and pH, 6.9. The soil has a sandy loam texture (sand 65.0%, silt 28.0%, clay 7.0%). The soil intended for our bioremediation studies has not been used for agricultural purposes during the past 5 years, located in Guangzhou, Southern China. To investigate the removal potential of bifenthrin by the yeast in soils, 200 g of soil were placed in 500 mL-Erlenmeyer flask and their water contents were adjusted to 40% of water-holding capacity [39]. Soil moisture content was maintained at a constant level throughout the experiment by addition of distilled water when necessary. Bifenthrin was added to a final concentration of 50 mg·kg^−1^ of soil in acetone solution. After mixing and solvent evaporation the microbial suspensions were inoculated into soils (in triplicate) by drip irrigation to give the final concentration of 1.0×10^7^ CFU·g^−1^ of soil and incubated at 32°C. The inoculum was thoroughly mixed under sterile conditions. In addition, sterilized soil (autoclaving at 121°C for 60 min) was also used to compare the removal efficiency of bifenthrin in soils [41], [42]. Triplicate samples of non-inoculated soils were kept as control. After 2, 4, 6, 8, and 10 days, soil samples (20 g) were removed for analysis to determine the residual concentration of bifenthrin. The extracting and analytical methods were analogous to those used in the liquid medium.

### Chemical analysis

The pyrethroid residues were determined on an Agilent 1100 HPLC system equipped with a Hypersil ODS2 C_18_ reversed phase column (4.6 nm×250 mm, 5 µm) with array detection from 190–400 nm (total scan) based on retention time and peak area of the pure standard [29]. In brief, 30-mL of cell-free medium was extracted using 60 mL of acetone/petroleum ether (1∶1, *v*/*v*) in an ultrasonic bath. After partitioning, the supernatants were passed through a 0.22 mm polytetrafluoroethylene (PTFE) membrane filter (Millipore, USA), and the filtrates were concentrated using rotary evaporator (Heidolph, Germany). Residues were dissolved in acetone and analyzed by HPLC. A mixture of acetonitrile and water (85∶15, *v*/*v*) was used as the mobile phase at a flow rate of 1.0 mL·min^−1^. The injection volume was 10 µL.

The metabolites of bifenthrin were identified on an Agilent 6890N/5975 GC-MS system equipped with auto-sampler, an on-column, split/splitless capillary injection system, and with HP-5MS capillary column (30.0 m×250 µm×0.25 µm) with array detection from 30–500 nm (total scan). The operating conditions were as follows: the column was held at 160°C for 5 min, ramped at 10°C·min^−1^ to 200°C (first ramp), held at 200°C for 1 min, ramped at 10°C·min^−1^ to 280°C (second ramp), and then held at at 280°C for 8 min [40]. The temperatures corresponding to transfer line and the ion trap were 280°C and 230°C, respectively, and the ionization energy was 70 eV. The injection volume was 1.0 µL with splitless sampling at 250°C. Helium was used as a carrier gas at a flow rate of 1.5 mL·min^−1^.

### Statistical analysis

Results were assessed by analysis of variance (ANOVA) and statistical analyses were performed on three replicates of data obtained from each treatment. The significance (*p*<0.05) of differences was treated statistically by one-, two-, or three-way ANOVA and evaluated by post hoc comparison of means using lowest significant differences (LSD) using SAS software packages.

### Ethics Statement

No specific permits were required for the described field studies. No specific permissions were required for these locations. We confirm that the location is not privately-owned or protected in any way. We confirm that the field studies did not involve endangered or protected species.

## Results

### Isolation and identification of the bifenthrin-degrading strain ZS-02

**Figure 1:**
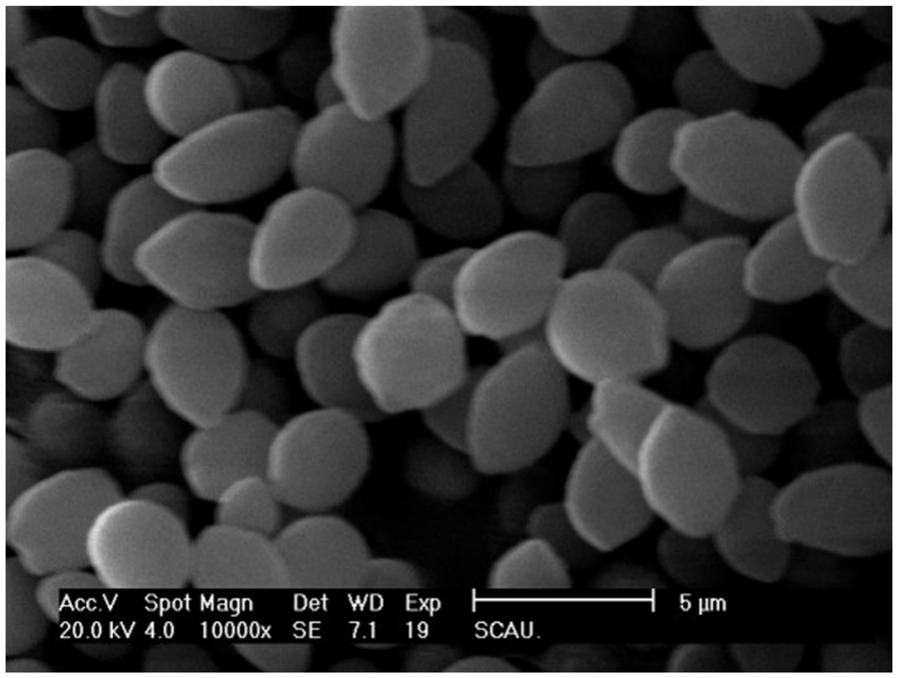
Morphological characteristics of strain ZS-02 under scanning electron microscope (10,000×).

Isolates were able to grow with bifenthrin as the sole carbon source identified one strain, designated ZS-02 and completely metabolized 50 mg·L^−1^ of bifenthrin within 8 days. This strain was an obligate aerobe (oxidase- and catalase-positive). Colonies were small, ivory, round, and with the entire margin when grown on YPD agar plates for 5 days. Cells were oval, and 3–4 µm in length and 2–3 µm in width (Fig. 1). It was positive in utilization of glucose, sucrose, trehalose, maltose, glycerol, xylose, raffinose, sorbierite, methyl-α-D-glucopyranoside, and cellobiose. It was negative in utilization of arabinose, adonitol, xylitol, galactose, inositol, 2-keto-D-gluconate, lactose, melezitose, and N-acetyl-D-glucosamine (Table 2).

**Table 2:** Morphological and physio-biochemical characteristics of *Candida pelliculosa* strain ZS-02.

Characteristics	Results	Characteristics	Results
Colonies	small, ivory, round, with entire margin	Cells	oval, 3–4 µm in length and 2–3 µm in width
Oxidase	+	Catalase	+
Glucose	+	Sucrose	+
Trehalose	+	Maltose	+
Glycerol	+	Xylose	+
Raffinose	+	Sorbierite	+
Methyl-α-D- glucopyranoside	+	N-acetyl-D- glucosamine	−
Cellobiose	+	Xylitol	−
Arabinose	−	Adonitol	−
Galactose	−	Inositol	−
Lactose	−	Melezitose	−
2-Keto-D-gluconate	−		

+, tested positive/utilized as substrate; −, tested negative/not utilized as substrate.

**Figure 2:**
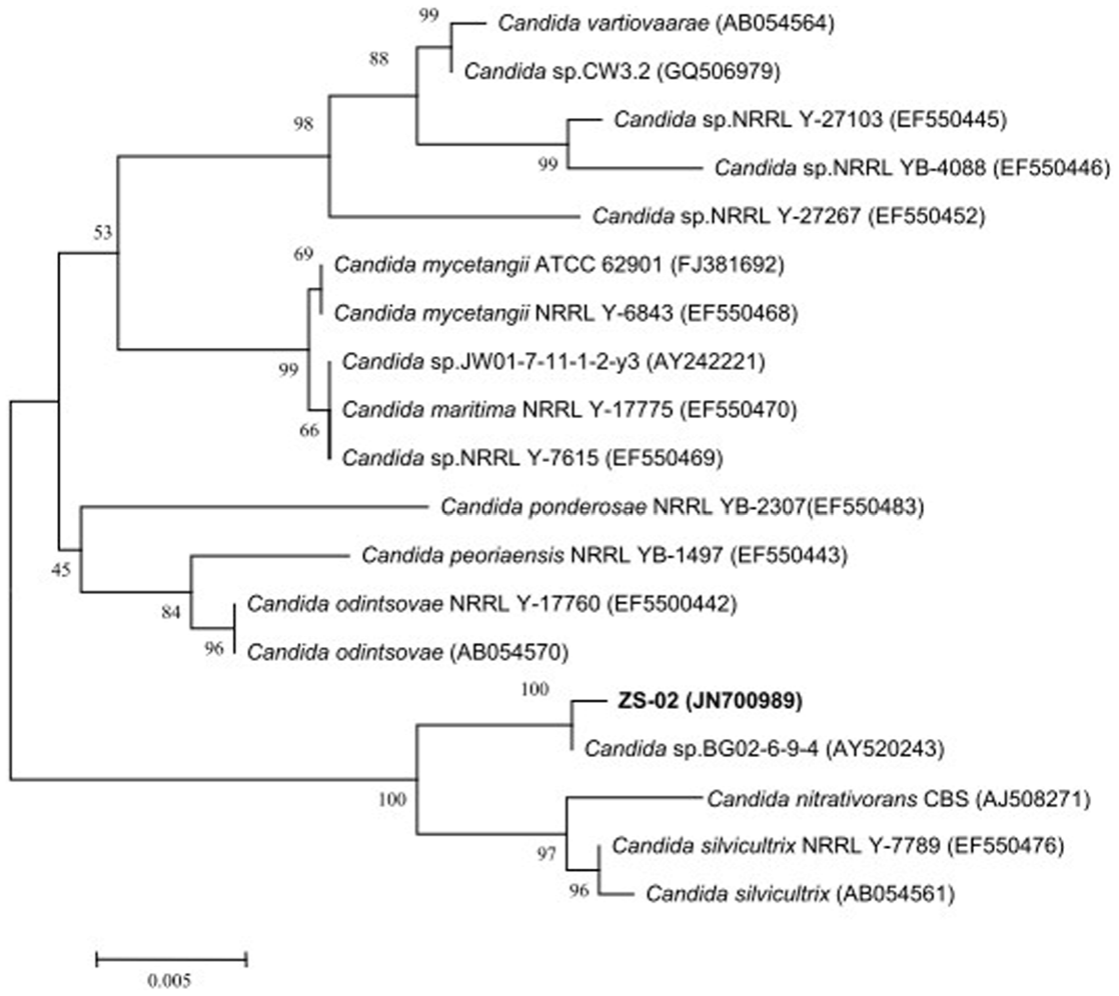
Phylogenetic tree based on the 18S rRNA sequence of strain ZS-02 and related strains. Numbers in parentheses represent the sequences accession number in GenBank. Numbers at the nodes indicate bootstrap values from the neighborhood-joining analysis of 1,000 resampled data sets. Bar represents sequence divergence.

PCR amplification of 18S rDNA gene from strain ZS-02, a single fragment of 1,452 bp, GenBank accession number JN700989, was obtained and completely sequenced. Phylogenetic analysis (Fig. 2) based on the 18S rDNA sequences revealed that strain ZS-02 belonged to *Candida* group and was closely related to *Candida* sp. BG02-6-9-4 (99%). The isolate was further classified as *Candida pelliculosa* by API 20C AUX system (bioMérieux, France), with very good identification (99.9%). Thus, strain ZS-02 was identified as *C. pelliculosa* based on morphological, physio-biochemical characteristics, API 20C AUX test, and 18S rDNA gene analysis. This is the first report of pyrethroid-degrading species from the genus *Candida*.

### Optimization of the bifenthrin-degrading conditions by strain ZS-02

The Box-Behnken design was applied to analyze the main and interactive effects of important variables including temperature (*X*_1_), pH (*X*_2_), and inoculum (*X*_3_) based on earlier single-factor experiments. The experimental design matrix and the response of dependent variable for bifenthrin degradation are presented in Table 1. Subsequently, the data from Table 1 were processed by response surface regression procedure of SAS software and results were fitted with the second-order polynomial model equation (Eq.(2)):
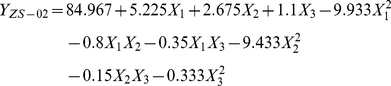
(2)where *Y*_ZS-02_ is the predicted bifenthrin degradation (%) by strain ZS-02; *X_1_*, *X_2_*, and *X_3_* are the coded values for the temperature, pH, and inoculum, respectively.

An *R* ^2^ of 0.9770 indicated that approximately 98% of the variability in response could be covered by the model, demonstrating that predicted values of the model were in perfect agreement with the experimental values. The results of the regression parameter estimate revealed that linear and square terms of temperature (*X*_1_) and pH (*X*_2_) values showed significant effects (*p*<0.05) on the bifenthrin degradation by strain ZS-02, but the linear and square terms of inoculum (*X*_3_) and the interaction terms were insignificant (*p*>0.05).

**Figure 3:**
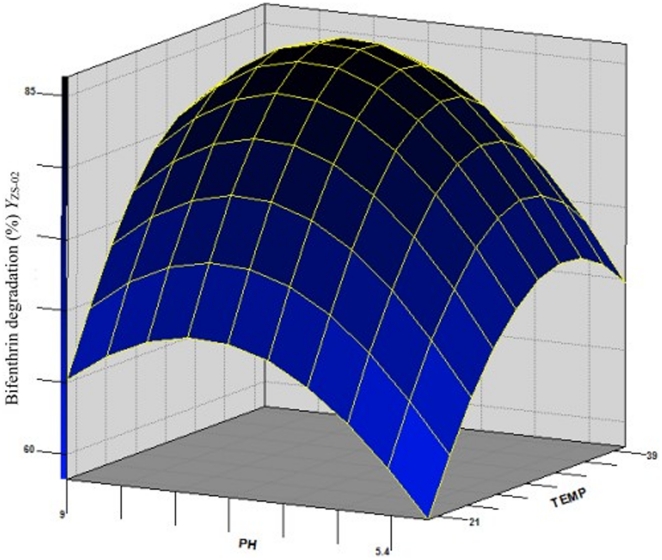
Response surface plot showing the effects of temperature and pH on bifenthrin degradation by strain ZS-02 with inoculum at OD_600_ = 0.3.

With the value of inoculum (the non-significant variable) fixed at OD_600 nm_ = 0.3, the three-dimensional response surface was plotted to directly display the effects of temperature and pH on the bifenthrin degradation by strain ZS-02 by day 5 (Fig. 3). As shown in Fig. 3, the plot of bifenthrin biodegradation had a theoretical maximum value of 86.56% at the stationary point. At the stationary point, the optimum levels for the two variables of *X_1_* and *X_2_* were found to be 0.23 and 0.12 in terms of the coded units, that is, temperature 32.3°C and pH 7.2, respectively. So the optimal conditions for bifenthrin degradation by strain ZS-02 were determined to be 32.3°C, pH 7.2, and inoculum at OD_600 nm_ = 0.3.

### Effect of extra carbon source on the growth of strain ZS-02 and degradation of bifenthrin in mineral salt medium (MSM)

**Figure 4:**
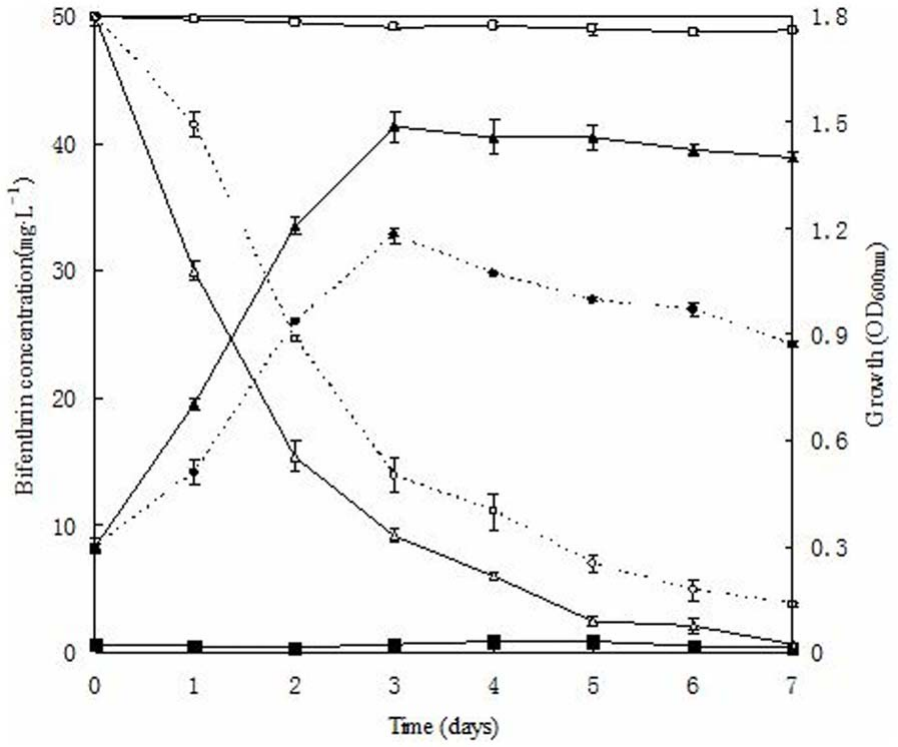
Cell growth of strain ZS-02 and degradation kinetics of bifenthrin during biodegradation studies. ○, degradation kinetics in MSM supplemented with bifenthrin as the sole carbon source; □, degradation kinetics in MSM supplemented with 1% glucose as an additional source of carbon; □, non-inoculated control (degradation); •, cell growth in MSM supplemented with bifenthrin as the sole carbon source; ▴, cell growth in MSM supplemented with 1% glucose as an additional source of carbon; ▪; non-inoculated control (growth). Values are the means of three replicates with standard deviation.

Growth of strain ZS-02 monitored by optical density (OD_600 nm_) and degradation kinetics of bifenthrin in MSM is shown in Fig. 4. After incubation for 7 days, 92.5% of the 50 mg·L^−1^ bifenthrin initially added to the medium was degraded by strain ZS-02, while in the same period disappearance rate of bifenthrin in MSM supplemented with 1% glucose was significantly higher (*p*<0.05) and reached 98.9%. Obviously, addition of carbon source enhanced the degradation of bifenthrin by this strain. No significant change in bifenthrin concentration was observed in non-inoculated cultures. Bifenthrin removal was associated with a concomitant increase of cell density. Similar to degradation, growth of strain ZS-02 was strongly stimulated in the presence of glucose, and was the most effective within 3 days of incubation whereas OD_600 nm_ of the culture was significantly increased from 0.3 to 1.5. In contrast, the OD_600 nm_ of the culture without additional of carbon source was significantly lower (*p*<0.05) and ranged from 0.3 to 1.0 after 3 days of incubation.

### Biodegradation of bifenthrin with different initial concentrations

**Figure 5:**
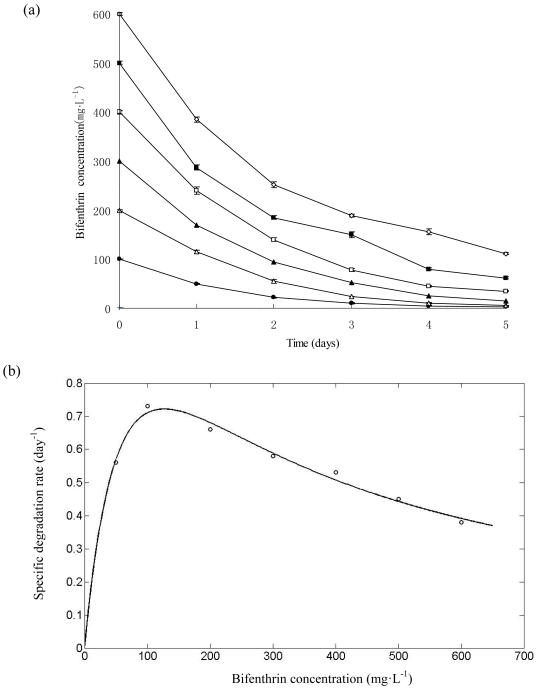
Biodegradation of bifenthrin with different initial concentrations. (a) Degradation kinetics of bifenthrin at different initial concentrations by strain ZS-02. •, 100 mg·L^−1^; □, 200 mg·L^−1^; ▴, 300 mg·L^−1^; □, 400 mg·L^−1^; ▪, 500 mg·L^−1^; □, 600 mg·L^−1^. Values are the means of three replicates with standard deviation. (b) Relationship between specific degradation rate and initial bifenthrin concentration by strain ZS-02.

Strain ZS-02 grew on bifenthrin up to the concentration, as high as 600 mg·L^−1^. As shown in Fig. 5a, the lag phase was extended at higher bifenthrin concentration. Strain ZS-02 completely degraded bifenthrin at concentration of 100 mg·L^−1^ within 5 days. At concentrations of 200, 300, and 400 mg·L^−1^, the degradation rates reached 97.1%, 95.8%, and 91.3% after 5 days of incubation, respectively. However, only 87.6% and 81.4% degradation were achieved at higher initial concentrations of 500 and 600 mg·L^−1^, respectively.

The decrease in the specific bifenthrin degradation rate with an increase in the initial bifenthrin concentration implied that bifenthrin acts as an inhibitor to strain ZS-02. Therefore, the substrate inhibition model (Eq.(3)) adapted from Wang et al. [43] was used to explain the degradation kinetics of bifenthrin by strain ZS-02.
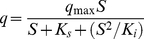
(3)where *q*_max_ is the maximum specific bifenthrin degradation rate (day^−1^), *K*_i_ is the substrate inhibition constant (mg·L^−1^), *K*_s_ is the half-saturation constant (mg·L^−1^), *S* is the substrate concentration (mg·L^−1^), and *S*_m_ is a critical inhibitor concentration of the substrate which decreases degradation.

The kinetic parameters of strain ZS-02 estimated from non-linear regression using matrix laboratory (MATLAB) software (Version 7.8) were *q_max_* = 1.7015 day^−1^ and *K*_s_ = 86.2259 mg·L^−1^. The inhibitory effect of bifenthrin was considered to occur in a linear manner at *K*_i_ = 187.2340 mg·L^−1^. The *S*_m_ was determined to be 127.06 mg·L^−1^. The relationship between *q* and *S* is shown in Fig. 5b. The value of *R* ^2^ was 0.9516 demonstrating that the experimental data were well correlated with the model. As indicated in Fig. 5b, when *S* were lower than 127.06 mg·L^−1^, *q* gradually increased. At higher concentrations, inhibition by bifenthrin became prominent.

### Biodegradation of various pyrethroids by strain ZS-02

**Figure 6:**
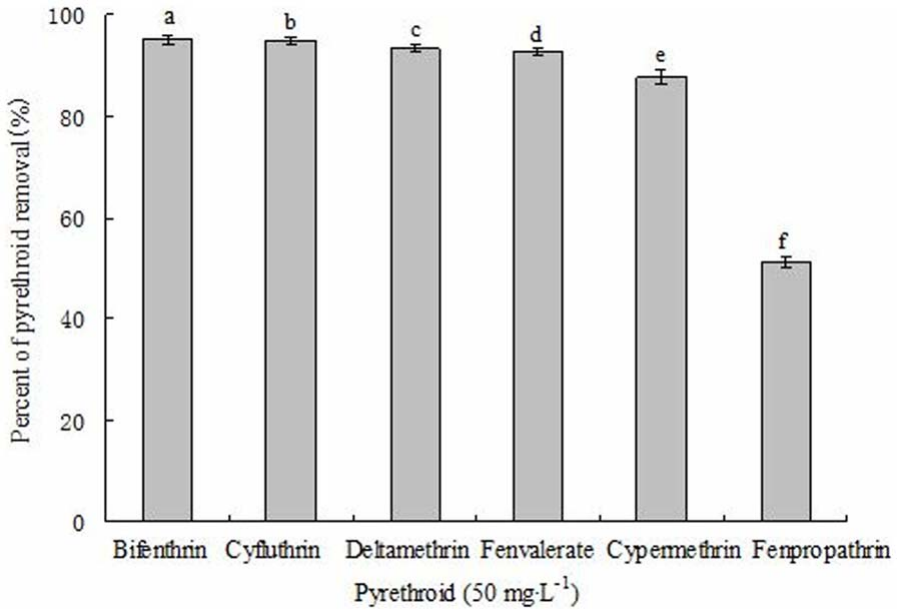
Degradation of various pyrethroids by strain ZS-02 by day 5. Values are the means of three replicates with standard deviation. Different letters indicate significant differences (*P*<0.05, LSD test).

The abilities of strain ZS-02 to degrade different pyrethroids are shown in Fig. 6. The strain was capable of metabolizing all of the pyrethroids tested. Bifenthrin was the most preferred substrate, with a degradation rate of 95.1% within 5 days of incubation. Cyfluthrin, deltamethrin, fenvalerate, cypermethrin, and fenpropathrin were degraded slower than bifenthrin, with degradation rates of 94.8%, 93.4%, 92.6%, 87.7%, and 51.3%, respectively.

### Identification of bifenthrin metabolites

To illuminate the pathway of bifenthrin degradation by strain ZS-02, bifenthrin metabolic products in cell-free filtrates were extracted and confirmed by GC-MS. The degradation products identified on the basis of mass spectrum were matched with authentic standard compounds from the National Institute of Standards and Technology (NIST, USA) library database.

The GC-MS analysis revealed the presence of five degradation products, as summarized in Table 3. The peak at retention time of 16.391 min corresponded to bifenthrin standard. This peak decreased over time and completely disappeared after 8 days. Bifenthrin [A] disappeared concomitantly with formation of five degradation products (Fig. S1). The five compounds corresponded with those of authentic cyclopropanecarboxylic acid [B], 2-methyl-3-biphenylyl methanol [C], 4-trifluoromethoxy phenol [D], 2-chloro-6-fluoro benzylalcohol [E], and 3,5-dimethoxy phenol [F]. The retention times of these compounds were 9.561, 9.508, 8.785, 11.327, and 12.674 min, respectively. However, the peaks corresponding to these compounds were transient and they disappeared finally. Eventually, no persistent accumulative product was detected.

**Table 3:** Chromatographic properties of metabolites of bifenthrin degraded by strain ZS-02.

Code	Retention time (min)	*m*/*z*	Compounds
A	16.391	458	Bifenthrin
B	9.561	195	Cyclopropanecarboxylic acid
C	9.508	282	2-Methyl-3-biphenylyl methanol
D	8.785	430	4-Trifluoromethoxy phenol
E	11.327	282	2-Chloro-6-fluoro benzylalcohol
F	12.674	281	3,5-Dimethoxy phenol

**Figure 7:**
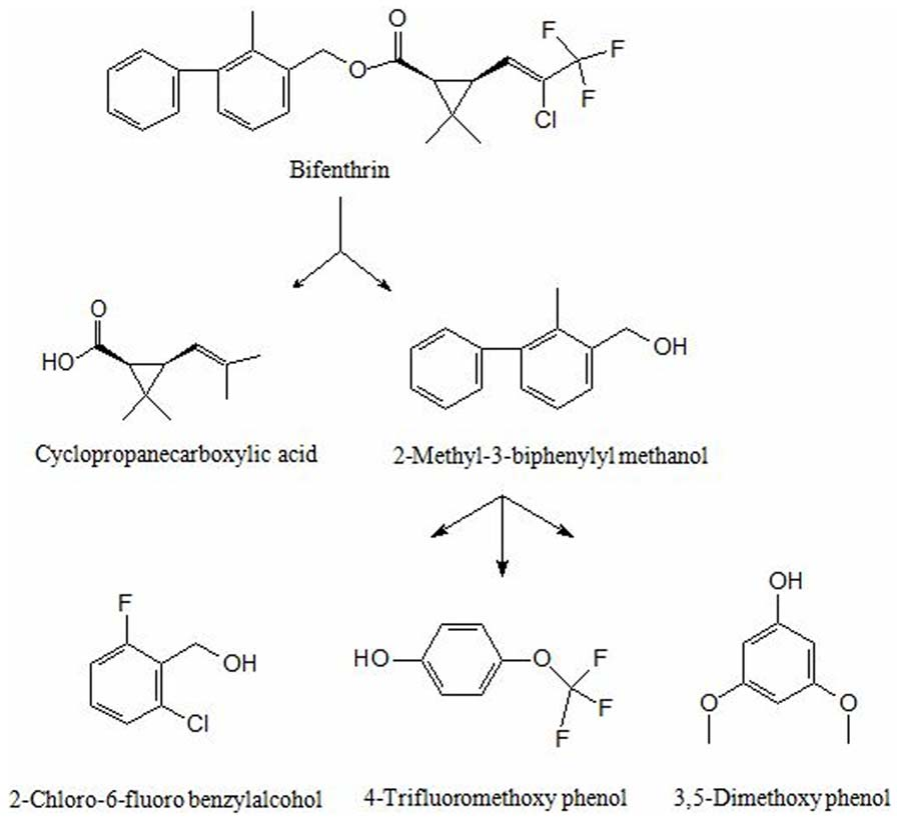
Proposed pathway for the degradation of bifenthrin by strain ZS-02.

On the basis of the metabolic products formed, we proposed the degradation pathway for bifenthrin by strain ZS-02 (Fig. 7). In other words, the parent bifenthrin [A] was first metabolized by hydrolysis of the carboxylester linkage to produce cyclopropanecarboxylic acid [B] and 2-methyl-3-biphenylyl methanol [C]. Subsequently, 2-methyl-3-biphenylyl methanol [C] was further transformed by biphenyl cleavage, resulting in formation of 4-trifluoromethoxy phenol [D], 2-chloro-6-fluoro benzylalcohol [E], and 3,5-dimethoxy phenol [F]. Finally, all these metabolites were not detected in the culture medium after 8 days of incubation. These results indicated that the added bifenthrin (50 mg·L^−1^) was completely degraded by strain ZS-02 without any accumulative metabolites at the end of reaction.

### Biodegradation of bifenthrin in soil

**Figure 8:**
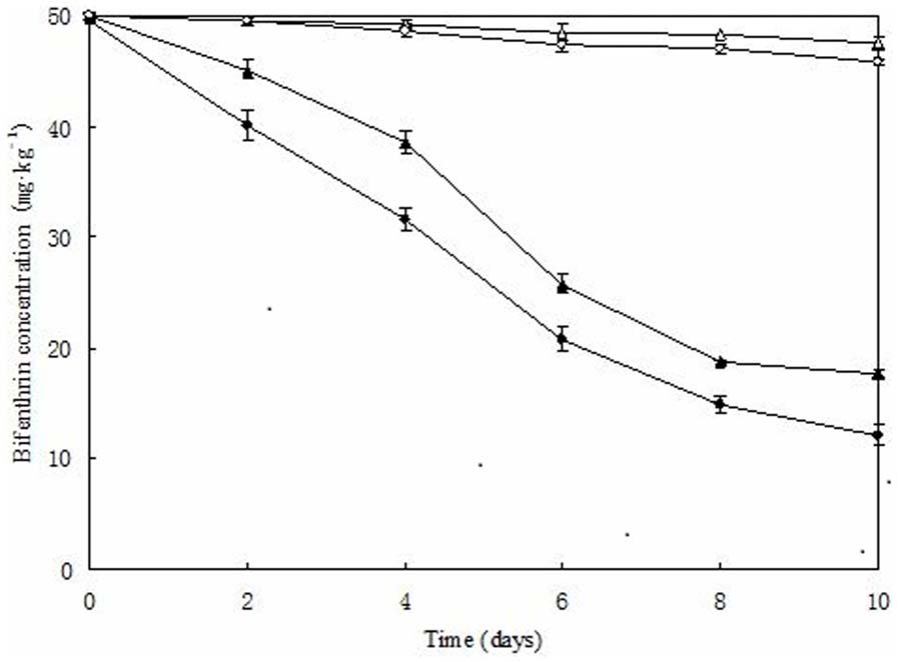
Degradation kinetics of bifenthrin in different contaminated soils by strain ZS-02. □, non-inoculated control (sterilized soil); ○, non-inoculated control with indigenous microbial community (non-sterilized soil); ▴, sterilized soil introduced with strain ZS-02; •, non-sterilized soil introduced with strain ZS-02. Values are the means of three replicates with standard deviation.

The potential of strain ZS-02 to eliminate bifenthrin in contaminated soils was investigated under controlled environmental conditions (temperature and soil humidity). The degradation kinetics are shown in Fig. 8. In the non-sterilized soil introduced with strain ZS-02 (1.0×10^7^ CFU·g^−1^ of soil), bifenthrin degradation started rapidly as utilization was observed at the beginning of incubation, apparently there was no lag phase and 75.1% of the 50 mg·kg^−1^ bifenthrin initially added to the soil was removed after 10 days of treatment; whereas in control with indigenous soil microorganisms, this activity decreased only by 8.4%. In case of sterilized soil inoculated with strain ZS-02, 64.7% of added bifenthrin was eliminated within 10 days; while a non-inoculated control showed that the total bifenthrin content decreased only by 3.4%. Obviously, strain ZS-02 could efficiently eliminate bifenthrin in contaminated soils as compared to the non-inoculated control. In addition, the disappearance rate of bifethrin in the non-sterilized soil was higher (*p*<0.05) than that in the sterilized soil.

To confirm the effects on degradation of bifenthrin in different soils by strain ZS-02, the first-order kinetic model (Eq.(4)) adapted from Cycoń et al. [39] was used to describe the rate of total bifenthrin reduction.
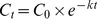
(4)where *C*_0_ is the initial concentration of bifenthrin at time zero, *C*_t_ is the concentration of bifenthrin at time *t*, *k* and *t* are the degradation rate constant (day^−1^) and degradation period in days, respectively.

The half-life (*t*_1/2_) for bifenthrin in various soils was calculated using the general formula as expressed in Eq.(5).
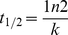
(5)where *k* is the rate constant (day^−1^).

The kinetic parameters for all runs calculated from the equations are tabulated in Table 4. Degradation kinetics showed that the process corresponds to first-order kinetics with *R* ^2^ ranging from 0.9492 to 0.9860, demonstrating that the experimental data were well correlated with the model. The kinetic constant (*k*) for the non-sterilized soil with strain ZS-02 was 0.1411 day^−1^, while the *k* for the sterilized soil was 0.1096 day^−1^. In the sterilized soil, longer half-lives (*t*_1/2_) of 6.3 days were observed, as compared to the non-sterilized soil with a *t*_1/2_ of 4.9 days. These results revealed that the bifenthrin removal efficiency by strain ZS-02 could be enhanced by the indigenous soil microorganisms. Furthermore, the *t*_1/2_ for bifenthrin in various soils introduced with strain ZS-02 (*t*_1/2_ = 4.9–6.3 days) were significantly shortened (*p*<0.05) as compared to that in the non-inoculated control soils (*t*_1/2_ = 78.8–130.7 days).

**Table 4:** Kinetic parameters of degradation of bifenthrin in soils by strain ZS-02.

Soil treatments	Regression equation	*k* (day^−1^)	*t*_1/2_ (days)	*R* ^2^
nSS+bifenthrin	*C_t_* = 50.1846e^−0.0088*t*^	0.0088	78.8	0.9799
SS+bifenthrin	*C_t_* = 50.0841e^−0.0053*t*^	0.0053	130.7	0.9806
nSS+bifenthrin+ZS-02	*C_t_* = 51.1033e^−0.1411*t*^	0.1411	4.9	0.9860
SS+bifenthrin+ZS-02	*C_t_* = 52.8446e^−0.1096*t*^	0.1096	6.3	0.9492

Each figure in the table represents the mean of three replicates. All figures were significantly different at *p*<0.05. nSS: non-sterilized soil with indigenous soil microbes; SS: sterilized soil without inoculum; +: introduced with; *C_t_*: residual concentration of bifenthrin (mg·kg^−1^); *t*: degradation period (days). *R* ^2^: correlation coefficient.

## Discussion

Bifenthrin, a broad-spectrum type I synthetic pyrethroid insecticide, has been heavily used throughout the world for pest control both in agricultural and urban environment during the past two decades, resulting in widespread contamination [2], [3], [5], [8], [16]. Bioremediation involves the use of living microorganisms to degrade and detoxify pollutants, and is generally considered to be the most significant process determining the fate and behavior of xenobiotics in the environment [44]. Although several microorganisms capable of degrading pyrethroids have been isolated from diverse geographic locations [26]–[30], yet no report on the isolation of bifenthrin-degrading strain is available. A possible reason was attributed to that this chemical is persistent and refractory to microbial degradation [45]. Additionally, the existing papers lack the information involved in the biodegradation of pyrethroids by yeast. Yeast possesses the biochemical and ecological capacity to degrade environmental organic chemicals, either by chemical modification or by influencing chemical bioavailability [46]. However, the potential use for yeast in bioremediation of pyrethroids has not received the attention it deserves. This is the first report to our knowledge about bioremediation of pyrethroids by yeast.

In the present study, the screening of bifenthrin-degrading yeast by the method of enrichment procedure from active sludge affected by pyrethroids allowed us to select some potential isolates with a high survivability in the environment and maximal degrading activity towards bifenthrin. It was generally considered that the conditions for environmental microorganisms enrichment and screening are crucial in the selection of isolates not only with the desired degrading enzyme systems but having specific regulation of the degradation pathways as well [47]. One most active yeast, designated ZS-02, able to completely metabolize bifenthrin was identified as *Candida pelliculosa* based on morphological, physio-biochemical characteristics, API 20C AUX test, and 18S rDNA gene analysis. Previous research has shown that the potential pyrethroid-degrading microorganisms were mostly from genera *Bacillus* and *Pseudomonas* [26], [27], [38], while *C. pelliculosa* strain ZS-02 appeared to be a new species that was found highly effective in degrading bifenthrin and several other pyrethroids. Members of this genus are ubiquitous in the environment and some isolates have antagonistic effect on pathogen due to their production of bioactive secondary metabolites [48]. However, there is limited information concerning the use of these isolates to degrade and detoxify pollutants. The current results expand their use.

Previous studies have shown that temperature and pH are two important factors which strongly influence the degradation ability of microorganisms able to degrade xenobiotic compounds [39], [49], [50]. Our results indicated that strain ZS-02 engaged in degrading bifenthrin over a wide range of temperatures (20–40°C) and pH (5–9) particularly at low pH. This is an important feature of a microorganism to be employed for bioremediation of variable environments [49]. Furthermore, the optimal conditions for bifenthrin degradation by strain ZS-02 were determined to be 32.3°C and pH 7.2 via response surface methodology (RSM). RSM is an efficient statistical technique that has been successfully applied to optimize degradation conditions for various microorganisms [30], [32], [33], [38], [51]. It is a faster and less expensive method for gathering research result than the conventional practice of one factor optimization [51]. Moreover, RSM can distinguish interaction effects from the effects of individual factors through appropriate construction of the process model [30], [38]. In our studies, a mathematical model was developed, and this model could be effectively used to predict and optimize the bifenthrin degradation conditions by strain ZS-02 within the limits of chosen factors.

*C. pelliculosa* strain ZS-02 utilized bifenthrin as the sole carbon for growth as well as co-metabolized it in the presence of extra carbon source, thus suggesting adaptation to oligotrophic and eutrophic conditions. However, this observation did not quite agree with Sørensen et al. [52] who reported isoproturon-degrading strain *Sphingomonas* sp. SRS2 was unable to grow on rich media, thus only adaptation to oligotrophic conditions. Furthermore, the growth of strain ZS-02 was stimulated and degradation of bifenthrin significantly enhanced in the presence of glucose in our studies (Fig. 4). This might be because of the cometabolism with additional carbon source lead to enhanced degradation of bifenthrin by strain ZS-02. Similar accelerated degradation in the presence of extra carbon source was observed by Anwar et al. [50] and Cycoń et al. [39] for different microorganisms degrading organophosphates.

It was noteworthy that this particular strain could tolerate and efficiently degrade bifenthrin up to the concentration, as high as 600 mg·L^−1^. However, the specific bifenthrin degradation rate decreased with an increase in the initial bifenthrin concentration (Fig. 5b). These findings indicated that increased bifenthrin concentration had a marked effect on degradation performance of strain ZS-02, but did not lead to complete inhibition. These results proved that strain ZS-02 was responsible for bifenthrin degradation. Another important feature which is worth mentioning is that this strain was capable of degrading a variety of of pyrethroids including bifenthrin, cyfluthrin, deltamethrin, fenvalerate, cypermethrin, and fenpropathrin, demonstrating that the pyrethroid hydrolase involved in degradation may have broad-spectrum substrate specificity. Bifenthrin was degraded faster than other pyrethroids tested, suggesting that presence of the cyano group caused relative reduction in the hydrolysis rate, due to either stabilization of the ester bond or toxic effect of the substituent group. Another possible reason for degradation reduction could be attributed to the fact that substitution of the biphenyl for a 3-phenoxybenzyl hinders the pyrethroid hydrolase-substrate interaction in strain ZS-02. However, the result contrasts with previous findings of Wang et al. [31] who reported that replacement of the 3-phenoxybenzyl with a biphenyl decreased the pyrethroid hydrolysis rate in *Ochrobactrum tritici* strain pyd-1. Additionally, cyfluthrin, deltamethrin, fenvalerate, and cypermethrin were degraded at much faster rates than fenpropathrin, indicating that with fluoro, bromo, chloroben, or chloro group on the chrysanthemic acid greatly improved the degradation efficiencies. It might be because of that these substituent groups strongly promoted the hydrolase activity. Therefore, we could conclude that the degradation efficiencies of strain ZS-02 depend on the molecular structure of the pyrethroids. Similar result has been proposed by Wang et al. [31], [45] and Guo et al. [29] for various microorganisms degrading pyrethroids.

It was generally suggested that ester hydrolysis by carboxylesterases was the main degradation pathway of pyrethroids in a multitude of species, from mammals, insects to microorganisms [28], [30], [38], [45], [53]. In our studies, the degradation pathway of bifenthrin by strain ZS-02 was investigated by metabolite identification, and the proposed pathway is shown in Fig. 7. It was evident from the results that strain ZS-02 first degraded bifenthrin by hydrolysis of ester linkage to produce cyclopropanecarboxylic acid and 2-methyl-3-biphenylyl methanol, leading to loss of its insecticide activity. Subsequently, 2-methyl-3-biphenylyl methanol was further transformed by biphenyl cleavage to form 4-trifluoromethoxy phenol, 2-chloro-6-fluoro benzylalcohol, and 3,5-dimethoxy phenol, resulting in its detoxification. Finally, all these degradation products were not detected by GC-MS after 8 days of incubation. These results indicated that the 50 mg·L^−1^ bifenthrin initially added to the medium was completely degraded by strain ZS-02 without any accumulative metabolites at the end of reaction. To the best of our knowledge, this is the first report of a novel pathway of degradation of bifenthrin by hydrolysis of ester linkage and cleavage of biphenyl in a microorganism. The bifenthrin metabolic pathway by ZS-02, however, appears to be similar to the initial step of cypermethrin degradation by *Micrococcus* sp. strain CPN 1, in which cypermthrin was converted to an acid and an alcohol by hydrolysis [28].

The potential of strain ZS-02 to eliminate bifenthrin in contaminated soils was evaluated to find out whether such strain is suitable for bioremediation purposes. In these experiments soils were inoculated with 1.0×10^7^ CFU·g^−1^ of soil and this inoculum density was found to efficiently remove bifenthrin from the environment. As shown in Fig. 8, the introduced strain ZS-02 quickly adapted to the environment and rapidly degraded bifenthrin at the beginning of incubation without any apparent lag phase. After 10 days of incubation, 75.1% and 64.7% of the added bifenthrin (50 mg·kg^−1^) was eliminated in the non-sterilized and sterilized soils, respectively. The *t*_1/2_ for bifenthrin was remarkably reduced by 63.9 and 124.4 days as compared to the control, implying that strain ZS-02 may have great potential to eliminate bifenthrin in contaminated soils. Furthermore, the removal efficiency of bifenthrin in the non-sterilized soil was significantly higher (*p*<0.05) than that in the sterilized soil, indicating that the indigenous soil microorganisms strongly promoted the ability of strain ZS-02 to eliminate bifenthrin. Enhancement of degradation could be explained by this reason that the introduced strain ZS-02 and soil microorganisms may have a synergistic effect on removal of pollutants. These results are consistent with previous observation [41], [42].

In conclusion, the *C. pelliculosa* strain ZS-02 isolated in the present study appeared to be highly efficient in degrading bifenthrin in different contaminated soil and water resources. Degradation of bifenthrin occurred at 20–40°C and pH 5–9. This is an important feature of a microorganism to be employed for bioremediation of variable environments. The yeast utilized bifenthrin as the sole carbon for growth as well as co-metabolized it in the presence of extra carbon source, thus suggesting adaptation to oligotrophic and eutrophic conditions. Another important feature which is worth mentioning is that this strain was proficient in degrading a variety of pyrethroids, indicating that the yeast could be ideal for remediation of soil and water contaminated with pyrethroids. Moreover, the yeast harbors the metabolic pathway for the detoxification of bifenthrin. This is the first report of a novel pathway of degradation of bifenthrin by hydrolysis of ester linkage and cleavage of biphenyl in a microorganism, which we propose plays an important role in the bifenthrin biogeocycle. Finally, strain ZS-02 is the first described yeast capable of degrading bifenthrin and several other pyrethroids.

## Supporting Information

Figure S1 **GC-MS spectra of metabolites produced from bifenthrin by** ***Candida pelliculosa*** **strain strain ZS-02.** (a) Bifenthrin; (b) Cyclopropanecarboxylic acid; (c) 2-Methyl-3-biphenylyl methanol; (d) 4-Trifluoromethoxy phenol; (e) 2-Chloro-6-fluoro benzylalcohol; (f) 3,5-Dimethoxy phenol.
(TIF)
