# Supplementary figures and images for: Microbial Detoxification of Bifenthrin by a Novel Yeast and Its Potential for Contaminated Soils Treatment
Source: PLoS One. 2012 Feb 13;7(2):e30862. doi: 10.1371/journal.pone.0030862 (PMC3278408; doi:10.1371/journal.pone.0030862)

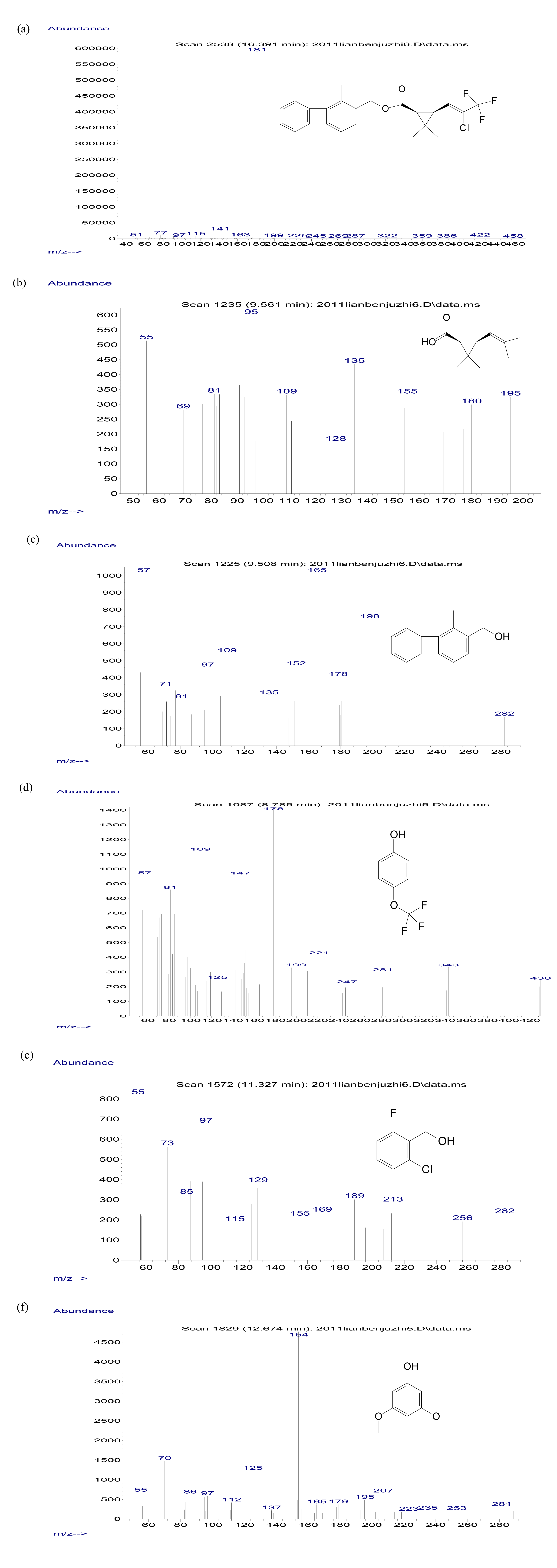

Supplement: Figure S1 — GC-MS spectra of metabolites produced from bifenthrin by Candida pelliculosa strain strain ZS-02. (a) Bifenthrin; (b) Cyclopropanecarboxylic acid; (c) 2-Methyl-3-biphenylyl methanol; (d) 4-Trifluoromethoxy phenol; (e) 2-Chloro-6-fluoro benzylalcohol; (f) 3,5-Dimethoxy phenol. (TIF) [file pone.0030862.s001.tif]
